# Provision of National Institute for Health and Care Excellence‐adherent cognitive behavior therapy for psychosis from inpatient to community settings: A national survey of care pathways in NHS mental health trusts

**DOI:** 10.1002/hsr2.198

**Published:** 2020-10-21

**Authors:** Pamela Jacobsen, Manting Tan

**Affiliations:** ^1^ Department of Psychology University of Bath Bath UK

## 1 INTRODUCTION

Guidelines from the United Kingdom's National Institute for Health and Care Excellence (NICE) for psychosis and schizophrenia (GC178, update 2014, https://www.nice.org.uk/guidance/cg178) recommend cognitive behavior therapy for psychosis (CBTp) be offered to all service users. The guidelines further state that CBTp “*can be started either during the acute phase or later, including in inpatient settings*” (guidelines 1.4.4.1). There is an additional recommendation that when CBTp is started within inpatient settings “*the full course should be continued after discharge without unnecessary interruption*” (guideline 1.4.4.5). However, it is not clear whether these guidelines are a good fit for how care pathways are currently configured in NHS mental health trusts. Trusts may not be able to offer continuity of care between inpatient and community settings for various reasons. For example, inpatient and community psychology services may be separate from each other, or there may be no CBTp provision at all within inpatient services. Although previous audits have found generally low levels of the provision of CBTp for service users with psychosis within NHS mental health trusts,^1^, ^2^, ^3^ no study has yet examined this issue of continuity of care between inpatient and community settings. This is despite evidence that the transition from inpatient to community care is a highly sensitive time in the care pathway, given the risk of untimely readmission to hospital^4^ and other adverse events, including risk of self‐harm and suicide.^5^ Qualitative studies have also highlighted the value that both staff and service users place on good continuity of care after an inpatient admission.^6^, ^7^

Our research question was therefore: What proportion of NHS mental health trusts in England have care pathways which allow for service users with psychosis to continue a full course of CBT which has been started within an inpatient setting, without interruption after discharge into the community, in line with NICE guidelines?

## 2 METHOD

Ethical approval for the study was given by the University of Bath Psychology Research Ethics Committee (Ref: 19‐319). The survey question below was distributed was via email to Heads of Psychological Therapies (or equivalent) at NHS mental health trusts in England. Healthcare is devolved in the other nations of the United Kingdom, hence our focus on England only.

Survey Question: Is it currently *possible* (on survey date of 1st Jan 2020) for service users with psychosis, to continue a full course of CBT which has been started within an inpatient setting, without interruption after discharge, anywhere in the Trust?

The email made it clear that participation was voluntary, and we were not asking for any data on numbers of service users who have received psychological therapies, as our question related only to care pathway configuration (see Data S1 for full wording). We asked for any additional contextual information the respondent was able to give to explain their answer. This was voluntary, however, as to reduce the burden of responding and to encourage a high response rate to the primary question, which could be a simple “yes/no” answer. If we did not receive a response to the initial email, we sent up to two further reminder emails at 2‐week intervals. Data collection took place between January 16, 2020 and March 23, 2020. We unfortunately had to terminate data collection prematurely due to the COVID‐19 outbreak before we were able to contact all trusts.

## 3 RESULTS

**Figure 1:**
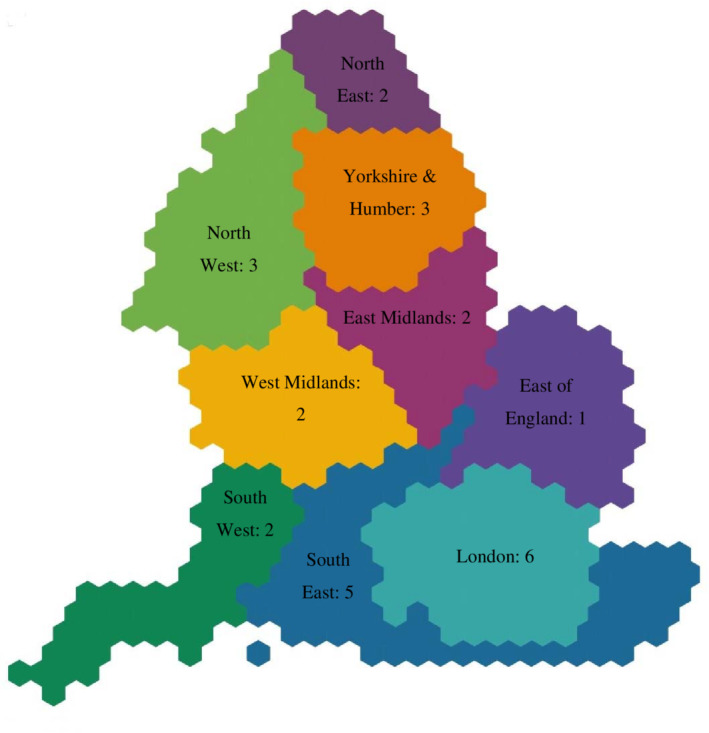
Numbers of NHS mental health trusts (*n* = 26) who responded to the survey in each geographical region of England

We identified 54 eligible NHS mental health trusts, which were operational on January 1, 2020. We sent the survey email to 41 trusts (41/54; 76% of eligible trusts) and received 26 responses (26/41; 63% response rate). In total, we received responses from 48% of all NHS mental health trusts in England (26/54). We received at least one response from all major geographical regions of England (Figure 1). One of the trusts who responded said they did not wish to answer the survey question, hence, we had available data for 25 trusts.

Approximately half of all trusts responded “yes” to the survey question (12/25; 48%). However, out of these who responded yes it was possible, some commented that it was not common practice due to limited staff resources (*n* = 5). Additionally, some trusts said it would only be possible for service users meeting criteria for early intervention (EI) services (*n* = 5). Some trusts gave additional information about how service configuration supported continuity of therapy provision. This was achieved in one of three main ways; (a) psychology staff had split inpatient/community posts (*n* = 3), (b) community staff could in‐reach to inpatient settings (*n* = 3), (c) inpatient staff could follow patients into the community to continue therapy which had started during an inpatient admission (*n* = 1).

For the 13 trusts who responded “no” to the survey question, the main reason given for this was that there was a separation between inpatient and community services, with no staff working across these settings in an integrated way (*n* = 9). Additional reasons given included CBTp not being offered at all within inpatient settings (*n* = 4), long waiting lists for therapy in the community (*n* = 2), and CBTp being mainly available in early intervention (EI) services (*n* = 1).

## 4 DISCUSSION

Out of the 25 trusts who responded to our survey, 48% reported that care pathways existed which made it possible for continuity of CBTp provision, in line with NICE guidelines. This finding means that in the majority of NHS mental health trusts, aspects of the NICE guidelines relating to the provision of CBTp are not a good fit for how services are currently configured. Furthermore, many of the trusts who said although it would be possible in theory to continue CBTp seamlessly from inpatient to community settings, it was not common practice. As all service users should be offered the best care in line with current guidelines, “common practice” is arguably a more important threshold, than whether a care pathway hypothetically exists, making these findings even more concerning. For trusts who answered “no” to the survey question, the main barrier reported was separation between inpatient and community services, so that the same staff members could not follow service users through this part of the care pathway.

The main limitation to our study is that we did not get data from 100% of all NHS mental health trusts in England. However, we obtained data from 25/54 trusts, which represents almost half of all NHS trusts. Furthermore, we were successful in obtaining data from a range of different trusts in terms of geographical location, urban/rural settings, and size of population served (Figure 1). As all of these variables may affect how well‐resourced trusts are to provide care in line with NICE guidelines, we believe it is reasonable to suggest our sample would be largely representative of the national picture. An additional limitation is that we acknowledge that NHS services are in a state of constant flux, with service reconfigurations a common occurrence. Hence, our results can only be considered a “snapshot” of current practice which is liable to change over time. Further research should focus on staff and service user views on the appropriateness of the NICE guidelines for psychosis with regards to continuity of care across inpatient and community settings, and whether it a current priority for them to improve compliance with this aspect of the guidelines. Previous surveys have shown that timely access to talking therapies is a priority for service users and their families.^8^ Data from CBTp trials also suggests that service users get most benefit from therapy, when it is completed as planned, rather than only being partially delivered,^9^ as may happen when the provision of therapy cannot bridge the gap between care settings. Therefore, we suggest further research on how care pathways are configured in health services, could help identify barriers and facilitators to wider implementation of evidence‐based psychological therapies. Further research could also explore if there are differences between provision of generic CBT, and CBT for psychosis as defined in the NICE guidelines, which would require a more in‐depth response from NHS managers in order to effectively delineate between generic and psychosis‐specific CBT approaches on offer.

## CONFLICT OF INTEREST

The authors declare no conflicts of interest.

## AUTHOR CONTRIBUTIONS

Conceptualization: Pamela Jacobsen

Formal analysis: Pamela Jacobsen, Manting Tan

Writing ‐ original draft preparation: Pamela Jacobsen, Manting Tan

Writing ‐ review and editing: Pamela Jacobsen, Manting Tan

  All authors have read and approved the final version of the manuscript.

  Pamela Jacobsen had full access to all of the data in this study and takes complete responsibility for the integrity of the data and the accuracy of the data analysis.

## TRANSPARENCY STATEMENT

Pamela Jacobsen affirms that this manuscript is an honest, accurate, and transparent account of the study being reported; that no important aspects of the study have been omitted; and that any discrepancies from the study as planned (and, if relevant, registered) have been explained.

## Supporting information


**Data S1**. Supporting Information.

## Data Availability

The data that support the findings of this study are available from the corresponding author upon reasonable request.
