# Supplementary material for: Provision of National Institute for Health and Care Excellence‐adherent cognitive behavior therapy for psychosis from inpatient to community settings: A national survey of care pathways in NHS mental health trusts
Source: Health Sci Rep. 2020 Oct 21;3(4):e198. doi: 10.1002/hsr2.198 (PMC7577664; doi:10.1002/hsr2.198)
Supplement: Supplementary file 1 — Data S1. Supporting Information. [file HSR2-3-e198-s001.docx]

**Supplementary Material: Email template send to NHS trusts**

Subject line: CBT for psychosis national survey

Dear….

**Re: A national survey of provision of CBT for psychosis from inpatient to community NHS settings**

We are conducting a survey across all NHS mental health trusts in England to find out more about provision of NICE-adherence psychological therapies for psychosis across acute pathways. We are contacting you in your capacity as lead for psychological therapies (or equivalent) in [INSERT NAME OF TRUST]

Currently, NICE guidelines for psychosis and schizophrenia (GC178, update 2014 https://www.nice.org.uk/guidance/cg178) recommend CBT be offered to all people, which “*can be started either during the acute phase or later, including in inpatient settings” (guidelines 1.4.4.1).* There is a further recommendation that when CBT is started within inpatient settings “*the full course should be continued after discharge without unnecessary interruption”* (guideline 1.4.4.5).

Our survey question is therefore:-

**Is it currently possible (on survey date of 1^st^ Jan 2020) for service users with psychosis, to continue a full course of CBT which has been started within an inpatient setting, without interruption after discharge, anywhere in the Trust?**

Please note we have purposely phrased the question to ask about whether this is ‘possible’ rather than whether this is common practice, or regardless of how many service users might receive therapy delivered in this way. This will help us identify whether the current NICE guidelines are a ‘good fit’ for how mental health services are currently configured across inpatient and community settings. We also recognise that service provision varies across different sites and services within the same Trust, hence we are asking whether this is possible anywhere in the Trust, rather than if it is possible in every service.

If you have any extra contextual information which would help us make sense of why you answered yes or no to the survey question, we would be very grateful to hear it as this will improve our interpretation of the survey responses overall. If you do not feel you are the best person to respond to this survey question, please forward to a colleague, or email us and pass on the appropriate contact details.

Please do not hesitate to get in touch if you have any questions or queries about the survey before making a response. This study was approved by the University of Bath Psychology Research Ethics Committee (Ref: 19-319).
